# HOXA-AS3 Promotes Proliferation and Migration of Hepatocellular Carcinoma Cells via the miR-455-5p/PD-L1 Axis

**DOI:** 10.1155/2021/9289719

**Published:** 2021-12-27

**Authors:** Cheng Zeng, Shaojun Ye, Yu Chen, Qu Zhang, Yan Luo, Liang Gai, Bo Luo

**Affiliations:** ^1^Zhongnan Hospital of Wuhan University, Institute of Hepatobiliary Diseases of Wuhan University, Transplant Center of Wuhan University, Hubei Key Laboratory of Medical Technology on Transplantation, Wuhan, China; ^2^The 3rd Xiangya Hospital of Central South University, Research Center of National, Health Ministry on Transplantation Medicine Engineering and Technology, Changsha, China; ^3^National Quality Control Center for Donated Organ Procurement, Hubei Clinical Research Center for Natural Polymer Biological Liver, Hubei Engineering Center of Natural Polymer-based Medical Materials, Wuhan, China; ^4^Reproductive Medicine Center, Wuhan Children's Hospital (Wuhan Maternal and Child Healthcare Hospital), Tongji Medical College, Huazhong University of Science and Technology, Wuhan, China; ^5^Department of Radiation Oncology, Hubei Cancer Hospital, Tongji Medical College, Huazhong University of Science and Technology, Wuhan, China

## Abstract

Hepatocellular carcinoma (HCC) is the most prevalent type of hepatic carcinoma. Long noncoding RNAs (lncRNAs) are considered crucial regulators of gene expression; however, their functions in HCC are not well understood. Thus, the present study is aimed at elucidating the functions of the lncRNA *HOXA-AS3* in HCC. The functions of the *HOXA-AS3*/miR-455-5p/programmed death-ligand 1 (*PD-L1*) axis were investigated *in vitro* via qRT-PCR and dual-luciferase reporter assays. The effect of *HOXA-AS3* expression on tumor growth and metastasis was assessed using a mouse xenograft model. High *HOXA-AS3* expression was observed in the HCC cell lines. Furthermore, overexpression of *HOXA-AS3* in HCC cells enhanced proliferation, migration, and invasion, regulated the cell cycle, and retarded apoptosis. We also identified an miR-455-5p binding site in *HOXA-AS3.* By sponging miR-455-5p, *HOXA-AS3* increased the expression of *PD-L1*. Additionally, both the inhibition of *PD-L1* and overexpression of miR-455-5p reversed the effects on cell proliferation and invasion triggered by the overexpression of *HOXA-AS3*. In conclusion, *HOXA-AS3* modulated the functions of HCC cells through the miR-455-5p/*PD-L1* axis. Therefore, *HOXA-AS3* may be a novel therapeutic target for HCC.

## 1. Introduction

Among hepatic carcinomas, hepatocellular carcinoma (HCC) is the most prevalent worldwide [1, 2]. The incidence of HCC is relatively low in the Western world, while there is a high prevalence in Asia. However, during the past 30 years, incidence has increased twofold in America and onefold in Britain [3, 4]. The 5-year survival of HCC patients remains low, and HCC causes approximately 600,000 annual deaths [5]. There has been little progress in developing effective treatments for HCC over the last 20 to 30 years. Therefore, there is an urgent need for new, reliable treatments for patients with HCC.

Long noncoding RNAs (lncRNAs) are a class of noncoding RNAs (ncRNAs) that exhibit a limited capacity for protein encoding [6]. Cumulative evidence has shown that lncRNAs can affect various biological processes and that they are involved in the generation and development of tumors [7, 8]. The most commonly explored role of lncRNAs is that of a major regulator of gene expression, which is performed by sequestering or “sponging” other regulators (e.g., miRNAs) [9]. For instance, lncRNA NR_027471 has been shown to inhibit the progression of osteogenic sarcoma as a competing endogenous RNA (ceRNA) of miRNA-8055 [10]. LINC01436 functions as a ceRNA that contributes to the progression of gastric cancer by sponging miR-513a-5p [11]. In other studies, various lncRNAs, including MALAT1 [12], LINC01189 [13], ST8SIA6-AS1 [14], and SNAI3-AS1 [15], have been shown to contribute to the pathophysiological aspects of HCC through competitive microRNA (miRNA) binding. Nevertheless, the functions of *HOXA-AS3* in HCC remain to be elucidated.

The aim of this study was to measure *HOXA-AS3* expression in HCC cell lines and analyze its effects. We hypothesized that *HOXA-AS3* promotes *PD-L1* expression by sponging miR-455-5p, thereby modulating HCC pathogenesis. Thus, *HOXA-AS3* may be a candidate therapeutic target for HCC.

## 2. Materials and Methods

### 2.1. Bioinformatics Analysis

The TCGA database (https://portal.gdc.cancer.gov/) was used for identifying differences in gene expression and overall survival. Lists of differentially expressed genes (*P* value < 0.05, |log2FC|  >  1) were prepared by using the limma package of R. Overall survival of HCC patients was prepared by using survival package and survminer package of R. The downloaded data from TCGA database can be found in Table S1. starBase 3.0 (http://starbase.sysu.edu.cn/) was used to predict miRNAs which have putative binding sites for HOXA-AS3 and PD-L1.

### 2.2. Culture of HCC Cells

HCC cell lines (Hep3B, SNU-387, Li-7, and HuH-7) and a normal human liver cell line (L-02) were purchased from the Shanghai Cell Bank of the Chinese Academy of Sciences (Shanghai, China). Cells were cultured at 37°C in an incubator with 5% CO_2_ in Dulbecco's modified Eagle's medium (DMEM) (Gibco BRL, Grand Island, NY, USA) with 10% fetal bovine serum (Gibco, Carlsbad, CA, USA).

### 2.3. Real-Time PCR, Cell Transfection, and Lentivirus Production and Transduction

Total RNA was extracted from HCC cells using TRIzol reagent (Invitrogen), following the manufacturer's instructions. To generate cDNA, the extracted total RNA was reverse transcribed using Takara's Reverse Transcription Kit. Then, a SYBR Green PCR kit (Takara, Dalian, China) was used for qPCR. GAPDH was used to normalize mRNA and lncRNA, and U6 was used to normalize miRNA. The primers used are listed in Table 1.

Anti-miR-455-5p, miR-455-5p mimics, anti-miR-NC, miR-NC, *HOXA-AS3* shRNA, and *HOXA-AS3-*expressing vectors for cell transfection were synthesized by Ruibo (Guangzhou, China). Cell transfection was performed using Lipofectamine 2000 reagent (Invitrogen) according to the manufacturer's instructions.

HEK293T cells were used to generate lentiviral particles with scrambled *HOXA-AS3* shRNA and *HOXA-AS3-*expressing vectors. Subsequently, recombinant lentiviruses were used to infect HCC cells, while 2 *μ*g/mL puromycin was used for cell selection.

### 2.4. Determination of Cell Proliferation

The CCK-8 (Beyotime, Beijing, China) was used to assess cell activity. Before adding the CCK-8 reagent, transfected cells in each well of a 96-well plate were subjected to overnight culture. A microplate reader was used to measure the optical density values at 450 nm.

Subsequently, a Cell-Light EdU Cell Proliferation/DNA Kit (RiboBio Co., Ltd., Guangzhou, China) was employed for the 5-ethynyl-2′-deoxyuridine (EdU) cell proliferation assay. In brief, cells were immobilized with 4% paraformaldehyde, stained with Apollo Dye Solution, incubated with EdU for 2 h, and mounted with Hoechst 33342. Then, a microscope was used to capture images, followed by counting of EdU-positive cells.

To assess the colony formation of HCC cells, monoplast suspensions of HuH-7 and Hep3B cells were seeded into each well of a 12-well plate at equal concentrations and incubated in DMEM containing 10% fetal bovine serum. After 12 days, visible colonies were stained and photographed for counting.

### 2.5. Transwell, Cell Cycle, and Apoptosis Assays

The upper transwell chamber was precoated with Matrigel for 30 min at 37°C, and 500 *μ*L of complete medium was added to the bottom chamber. Cells were inoculated into the upper chamber. After 48 h of incubation, cells in the bottom chamber were rinsed with PBS, immobilized with 4% paraformaldehyde, stained with crystal violet, and imaged with a microscope. Cell analysis was performed three times for each group.

Cell trypsinization was then conducted for separation, followed by rinsing twice with ice-cold PBS and immobilization with 70% ethanol overnight at -20°C. The next day, 100 *μ*g/mL RNase A (KeyGen BioTECH) and 50 *μ*g/mL propidium iodide were used for cell suspension, followed by incubation for 40 min at room temperature. Finally, the specific stages of the cell cycle were detected by flow cytometry.

For apoptosis assays, cells were rinsed with PBS and stained using the Annexin V-FITC Apoptosis Detection Kit (Affymetrix eBioscience) according to the manufacturer's instructions. Apoptosis was assessed using a FACS flow cytometer (BD Biosciences).

### 2.6. Dual-Luciferase Reporter Assay

The 3′-UTRs of *PD-L1* and *HOXA-AS3* were amplified and separately cloned downstream of the firefly luciferase gene in the pGL3 vector (Promega). These were called wild-type (WT) 3′-UTRs. Following the manufacturer's instructions, a QuickChange site-directed mutagenesis kit (Stratagene, Cedar Creek, USA) was employed for mutation induction, and mutant miR-455-5p binding sites were identified in the 3′-UTRs of both *PD-L1* and *HOXA-AS3*. The mutant 3′-UTRs were called MUT 3′-UTRs. WT or MUT 3′-UTRs of *PD-L1* and *HOXA-AS3* and miR-NC or miR-455-5p were used to transfect HCC cells. After 48 h, luciferase assays were performed using a dual-luciferase reporter assay system (Promega). The analysis was repeated three times per group.

### 2.7. Immunohistochemistry (IHC)

Using formalin-fixed and paraffin-embedded xenograft tumor tissue sections, anti-Ki-67 (ab16667; Abcam, Shanghai, China) and anti-PD-L1 antibodies (ab205921; Abcam) were used for IHC as reported previously [16, 17].

### 2.8. Tumor Formation *In Vivo*

After stable transfection, BALB/c (nu/nu) mice (5 weeks old) were administered 2 × 10^6^ Hep3B cells (*HOXA-AS3* or Lv-NC) by subcutaneous flank injection. Before euthanasia, the tumor volume (V) was determined weekly for four weeks, calculated as *V* = 0.5 × length × width^2^. After approximately 28 days, the mice were sacrificed by cervical dislocation to harvest the tumors surgically. Subsequently, the tumor tissues were photographed, weighed, and stored in liquid nitrogen before use. The *in vivo* experiments were performed at the SPF Animal Laboratory at Tongji Medical College, Huazhong University of Science and Technology. Experimental procedures involving animals obtained approval from the Ethics Committee for Experimental Animals of Hubei Cancer Hospital, Tongji Medical College, Huazhong University of Science and Technology.

An advanced-stage pulmonary metastasis model was constructed by inoculating each mouse with Hep3B cells (1 × 10^7^) through stable injection into the tail vein. After 4 weeks, mice were euthanized for lung collection and hematoxylin-eosin staining.

### 2.9. Bioinformatics Analysis and Statistical Analysis

The TCGA (https://portal.gdc.cancer.gov/) was used for identifying differences in gene expression. Lists of differentially expressed genes (*P* value < 0.05, |log2FC|  >  1) were prepared by using the limma package of R. Data are reported as mean ± standard deviation (SD). A two-tailed Student's *t*-test was used for comparisons between two groups, and one-way ANOVA was used for comparisons among multiple groups. The statistical significance threshold was set at *P* < 0.05.

## 3. Results

### 3.1. *HOXA-AS3* Expression Was Increased in HCC Cells

**Figure 1:**
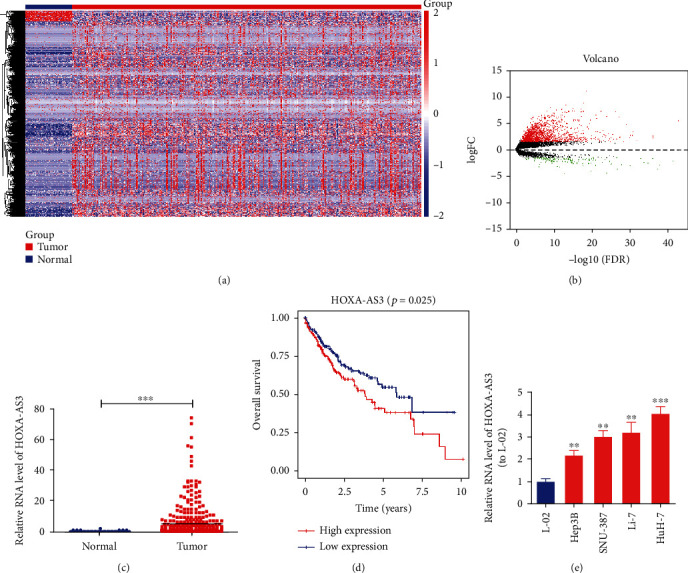
*HOXA-AS3* expression is increased in HCC cells. (a, b) TCGA heat map (a) and volcano map (b) of lncRNA expression in HCC patients. (c) *HOXA-AS3* expression in HCC tissues and controls in TCGA dataset. (d) Association of high *HOXA-AS3* expression with shorter overall survival in TCGA dataset. (e) *HOXA-AS3* expression in HCC cell lines. Data are reported as the mean ± SD of three separate experiments; ^∗∗^*P* < 0.01 and ^∗∗∗^*P* < 0.001.

TCGA database was used to select lncRNAs associated with HCC. We observed upregulated *HOXA-AS3* expression in HCC tissues (Figures 1(a)–1(c)), which was associated with unfavorable prognosis in HCC patients (Figure 1(d)). qRT-PCR was used to measure the *HOXA-AS3* expression level in a normal human liver cell line (L-02) and HCC cell lines (Hep3B, SNU-387, Li-7, and HuH-7). The findings revealed that HCC cells had considerably higher *HOXA-AS3* expression levels than L-02 cells (Figure 1(e)).

### 3.2. *HOXA-AS3* Promoted HCC Proliferation and Invasion *In Vitro*

**Figure 2:**
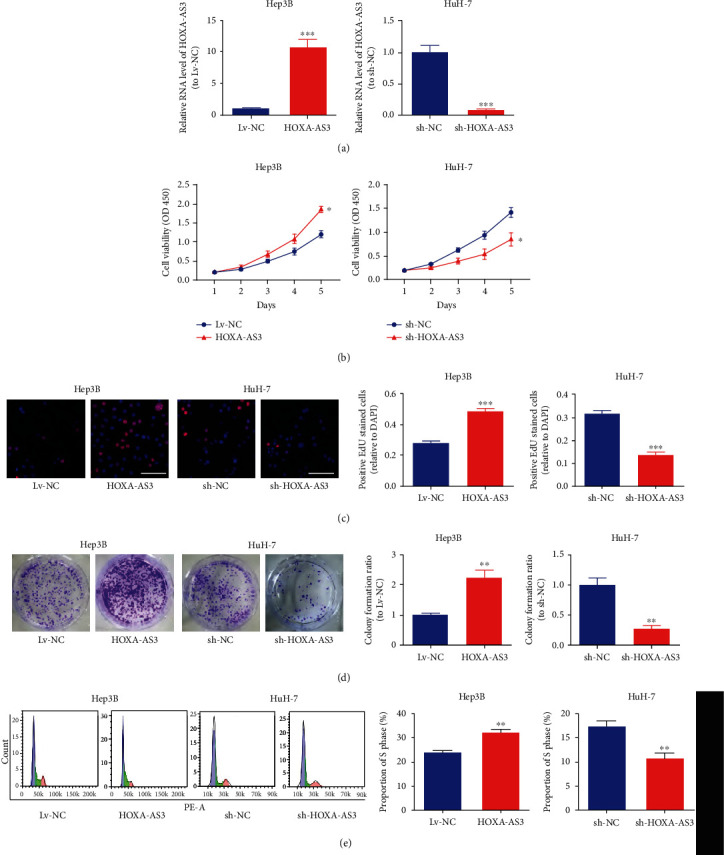
*HOXA-AS3* promotes HCC cell proliferation and cell cycle. (a) *HOXA-AS3* expression in transfected Hep3B and HuH-7 cells. (b) CCK-8 assay, (c) EdU (bar = 100 *μ*m), and (d) colony formation assays to determine the effects of *HOXA-AS3* on HCC cell proliferation. (e) Cell cycle of transfected Hep3B and HuH-7 cells. Data are reported as the mean ± SD of three separate experiments; ^∗^*P* < 0.05, ^∗∗^*P* < 0.01, and ^∗∗∗^*P* < 0.001.

**Figure 3:**
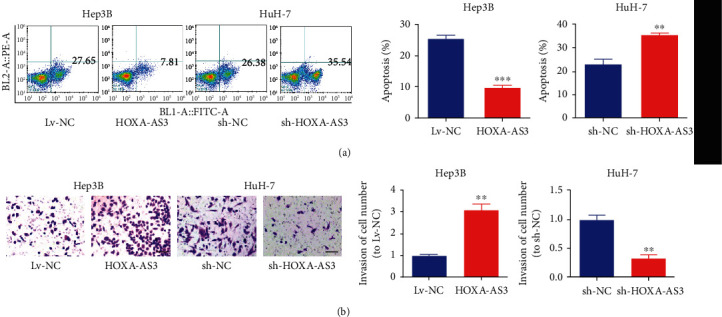
*HOXA-AS3* upregulation promotes HCC cell invasion but inhibits cell apoptosis. (a) Apoptosis assay showing higher *HOXA-AS3* level inhibits the apoptosis of HCC cells. (b) Transwell assay showing higher *HOXA-AS3* level enhances the invasion of HCC cells (bar = 100 *μ*m). Data are reported as the mean ± SD of three separate experiments; ^∗∗^*P* < 0.01 and ^∗∗∗^*P* < 0.001.

To determine how *HOXA-AS3* functions in HCC cells, we performed a variety of *in vitro* assays in Hep3B and HuH-7 cells. We overexpressed *HOXA-AS3* in Hep3B cells and downregulated *HOXA-AS3* expression in HuH-7 cells (Figure 2(a)). CCK-8, EdU, and colony formation assays showed that higher *HOXA-AS3* expression remarkably enhanced colony formation and cell proliferation (Figures 2(b)–2(d)). Furthermore, flow cytometry revealed that the S phase in the *HOXA-AS3* overexpression group was longer than that in the Lv-NC group (Figure 2(e)). Subsequently, apoptosis and transwell invasion assays, respectively, suggested that *HOXA-AS3* retarded HCC cell apoptosis (Figure 3(a)) and promoted HCC cell invasion (Figure 3(b)).

### 3.3. *HOXA-AS3* Promoted *PD-L1* Expression by Binding to miR-455-5p

**Figure 4:**
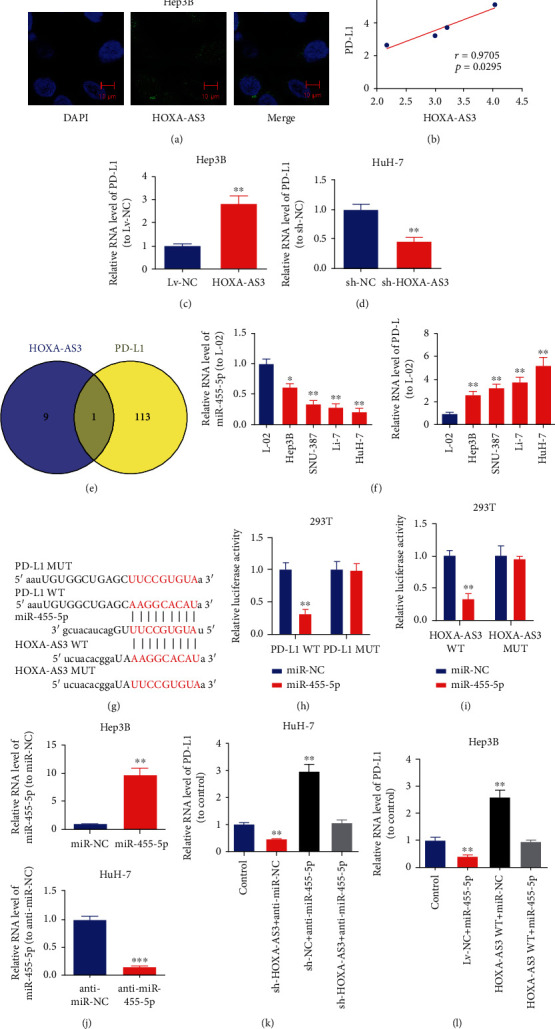
*HOXA-AS3* enhances *PD-L1* expression via binding to the 3′-UTR of miR-455-5p. (a) Typical images of the subcellular localization of *HOXA-AS3* in Hep3B cells as shown by RNA-FISH. (b) Association between *HOXA-AS3* expression and *PD-L1* expression in HCC cells evaluated by Pearson's correlation analysis. (c, d) Effects of *HOXA-AS3* overexpression or knockdown on *PD-L1* expression in HCC cells. (e) Bioinformatics analysis by using starBase. (f) Levels of miR-455-5p and *PD-L1* in HCC cells. (f–i) Dual-luciferase reporter assay with binding sites. (j) Effects of miR-455-5p mimics or inhibitor on miR-455-5p expression in HCC cells. (k) Effects of anti-miR-455-5p and *HOXA-AS3* shRNA on *PD-L1* expression in HCC cells. (l) Overexpression of a fragment of *HOXA-AS3* carrying the MRE of miR-455-5p could reverse the inhibition of *PD-L1* expression induced by overexpressed miR-455-5p. Data are reported as the mean ± SD of three separate experiments. ^∗^*P* < 0.05, ^∗∗^*P* < 0.01, and ^∗∗∗^*P* < 0.001.

First, the subcellular localization of *HOXA-AS3* was detected using RNA-FISH. Most *HOXA-AS3* was localized in the cytoplasm, with very little *HOXA-AS3* in the nucleus (Figure 4(a)). In previous studies, *PD-L1* was associated with unfavorable prognosis in HCC patients [18] and shown to contribute to HCC proliferation and metastasis [19]. Here, a positive correlation was observed between *HOXA-AS3* and *PD-L1* expression in HCC cells (Figure 4(b)). Furthermore, enhanced *HOXA-AS3* expression increased *PD-L1* expression in Hep3B cells (Figure 4(c)), and downregulation of *PD-L1* was observed after knockdown of *HOXA-AS3* in HuH-7 cells (Figure 4(d)).

Subsequently, bioinformatics analysis was conducted using starBase 3.0 (http://starbase.sysu.edu.cn/). Only miR-455-5p was found to have putative binding sites for both *HOXA-AS3* and *PD-L1* (Figure 4(e)). The qRT-PCR results showed that compared with the L-02 cell line, HCC cell lines exhibited notably lower miR-455-5p expression and higher *PD-L1* expression (Figure 4(f)). Bioinformatics analysis revealed a complementary relationship between the miR-455-5p sequence and sequences in the 3′-UTRs of both *PD-L1* and *HOXA-AS3* (Figure 4(g)).

Luciferase activity in HEK293T cells cotransfected with *PD-L1* WT 3′-UTR and miR-455-5p mimic was notably suppressed compared to that in cells cotransfected with *PD-L1* MUT 3′-UTR and miR-455-5p mimic (Figure 4(h)). HEK293T cells cotransfected with *HOXA-AS3* WT 3′-UTR and miR-455-5p mimic significantly inhibited luciferase activity, while cotransfecting mutant-type PD-L1 and miR-455-5p mimics did not affect the luciferase activity (Figure 4(i)).

Subsequently, we explored whether the modulation of *PD-L1* expression by *HOXA-AS3* in HCC cells was dependent on miR-455-5p expression. The transfection efficiency of miR-455-5p mimics and inhibitors is shown in Figure 4(j). In HCC cells, *PD-L1* expression was upregulated by downregulating miR-455-5p (Figure 4(k)). Additionally, the inhibition of *PD-L1* expression induced by *HOXA-AS3* shRNA was reversed by the inhibition of miR-455-5p expression (Figure 4(k)). Thereafter, we synthesized a plasmid (*HOXA-AS3* WT) that overexpressed a fragment of *HOXA-AS3* containing the MRE for miR-455-5p to analyze the effects of *HOXA-AS3* on *PD-L1* expression in cells with or without miR-455-5p. *HOXA-AS3* WT reversed the miR-455-5p mimic-induced suppression of *PD-L1* expression (Figure 4(l)), indicating that *HOXA-AS3* served as a sponge for miR-455-5p. In other words, when *HOXA-AS3* binds to the 3′-UTR of miR-455-5p, less miR-455-5p can bind to *PD-L1*, thus suppressing its translation. It is assumed that this is the mechanism underlying the effect of *HOXA-AS3* on *PD-L1* expression.

### 3.4. Increase in *HOXA-AS3* Expression Promoted Tumor Growth and Metastasis

**Figure 5:**
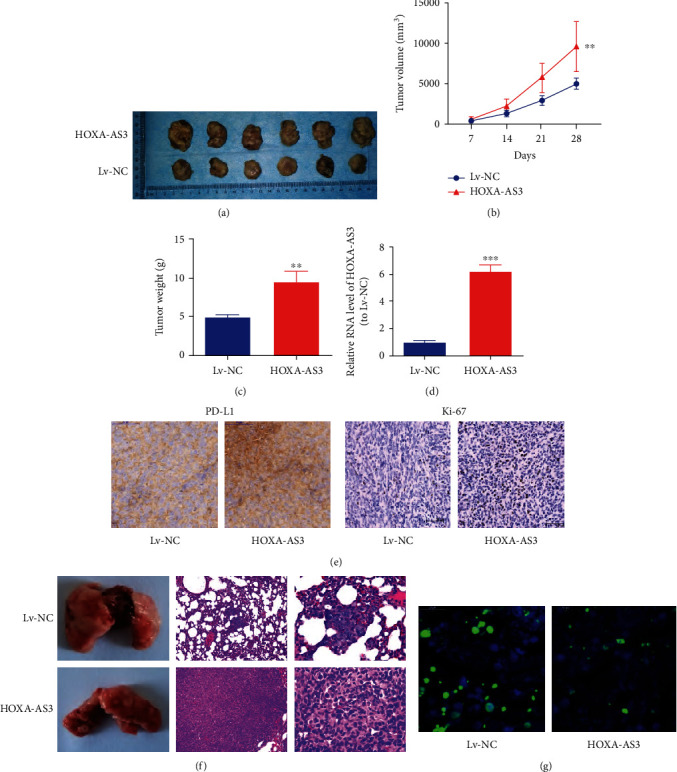
*HOXA-AS3* overexpression promotes the tumor growth and metastasis in vivo. (a) Xenograft tumors. (b) Faster growth of xenograft tumors from *HOXA-AS3* cells vs. tumors from Lv-NC cells. (c) Xenograft tumor weight. (d) Detection of *HOXA-AS3* expression in xenograft tumors. (e) Overexpressed *HOXA-AS3* notably contributes to *PD-L1* and Ki-67 levels in tumors vs. the negative control group (bar = 50 *μ*m). (f) Upregulation of *HOXA-AS3* contributes to tumor metastasis *in vivo*. Typical microscopic and macroscopic images of lungs (H&E staining). (g) Upregulation of *HOXA-AS3* represses apoptosis (bar = 20 *μ*m). ^∗∗^*P* < 0.01 and ^∗∗∗^*P* < 0.001.

We further explored whether higher *HOXA-AS3* expression contributed to tumor growth *in vivo*. Xenograft tumor growth increased with the overexpression of *HOXA-AS3* (Figure 5(a)). Furthermore, the mean weight and volume of xenograft tumors in the *HOXA-AS3* overexpression group were greater than that in the Lv-NC group (Figures 5(b) and 5(c)). Next, *HOXA-AS3* expression in xenograft tissues was analyzed (Figure 5(d)). IHC analysis indicated that the *HOXA-AS3* overexpression group also had higher Ki-67 and *PD-L1* expression than the Lv-NC group (Figure 5(e)). Additionally, in the *in vivo* lung metastasis model, overexpression of *HOXA-AS3* considerably enhanced lung metastasis (Figure 5(f)). Finally, TUNEL staining revealed that cell apoptosis was remarkably repressed by overexpression of *HOXA-AS3* (Figure 5(g)).

### 3.5. *HOXA-AS3* Regulated the Proliferation, Invasion, Apoptosis, and Cell Cycle of HCC Cells via the miR-455-5p/*PD-L1* Axis

**Figure 6:**
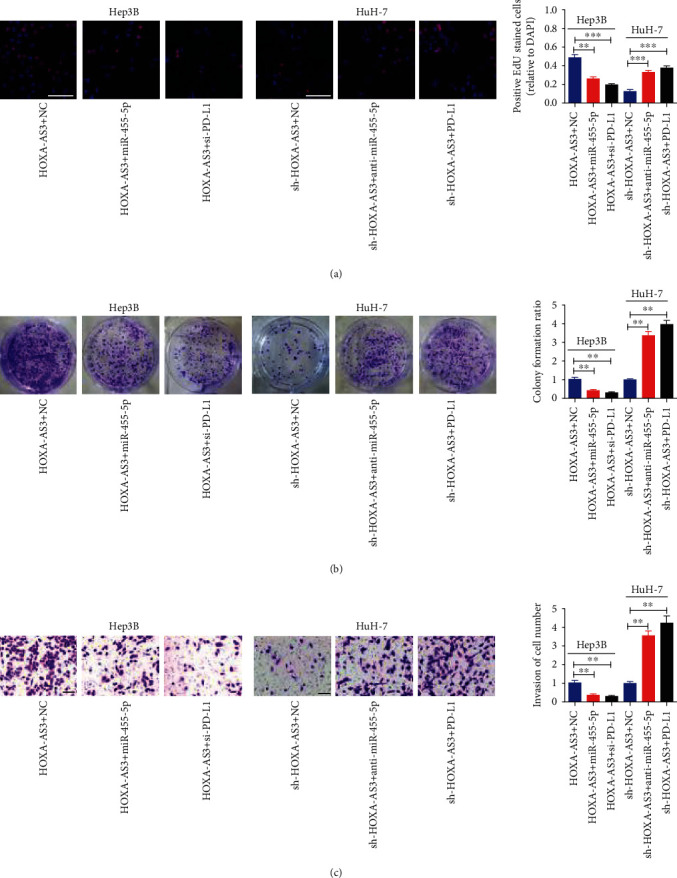
*HOXA-AS3* modulates HCC cell proliferation and invasion via miR-455-5p/*PD-L1* axis. (a) EdU (bar = 100 *μ*m), and (b) colony formation assays assessing the proliferation and colony formation of HuH-7 and Hep3B cells. (c) Images of HuH-7 and Hep3B cells by invasion assay (bar = 100 *μ*m). Data are reported as the mean ± SD of three separate experiments; ^∗∗^*P* < 0.01 and ^∗∗∗^*P* < 0.001.

To confirm the roles of the *HOXA-AS3*/miR-455-5p/*PD-L1* axis in HCC, rescue experiments were performed in HuH-7 and Hep3B cells. HuH-7 cells were transfected with the NC vector or *PD-L1* overexpression vector, and Hep3B cells were transfected with si-*PD-L1* or si-NC (Figure S1A). We then conducted colony formation, EdU, transwell invasion, apoptosis, and cell cycle assays, which suggested that overexpression of both anti-miR-455-5p and *PD-L1* reversed the effects of sh-*HOXA-AS3* in HuH-7 cells (Figures 6(a)–6(c), Figure S1B, 1C). Furthermore, miR-455-5p mimics and si-*PD-L1* reversed the impact of *HOXA-AS3* overexpression in Hep3B cells (Figures 6(a)–6(c), Figure S1B, 1C).

## 4. Discussion

Despite rapid development in strategies for the early diagnosis and treatment of HCC, the majority of patients develop metastasis and chemical resistance [20, 21]. To improve the prognosis of HCC patients, it is crucial to discover new therapeutic targets and improve our understanding of the pathways associated with cancer occurrence and progression [22, 23]. lncRNAs have been recently shown to have vital effects on the progression of various tumors, including HCC [24, 25]. Therefore, in this study, a dataset from TCGA was used to analyze HCC-related lncRNAs. *HOXA-AS3* was chosen as the subject, and its expression was evaluated in HCC cells.

Our assays indicated that *HOXA-AS3* contributed to cell proliferation and invasion and repressed apoptosis to a certain extent. In addition, overexpressed *HOXA-AS3* was shown to enhance the cell cycle transition from G1 to S phase. Moreover, the high *HOXA-AS3* expression group had shorter overall survival than the low *HOXA-AS3* expression group. These results suggest that *HOXA-AS3* is a candidate biomarker for the prognosis of patients with HCC and that it may promote HCC progression.

Tumor cells are known to alter T cell activities to avoid antitumor immune responses and ensure their survival [26, 27]. Importantly, tumor cells interact with and induce apoptosis of CD8+ T cells to contribute to tumor growth and metastasis, as shown in previous studies [28, 29]. Additionally, strong evidence from several studies has revealed that blocking PD-1/PD-L1 by neutralizing PD-1 or PD-L1 can abrogate the immune evasion of tumor cells and activate CD8+ T cells [30–32], indicating that antibodies against PD-1 and PD-L1 are effective clinical immunotherapies for cancers [33–35]. Studies have been conducted on the addition of anti-PD-L1 and anti-PD-1 antibodies to lymphoma immunotherapy [36, 37]. In other studies, PD-L1 knockdown was shown to suppress cell proliferation and invasion in head and neck cancer [38], ovarian cancer [39], and breast cancer [40]. Zhou et al. [41] revealed that LINC00473, acting as a sponge of miRNA-195-5p, drove the development of pancreatic cancer by increasing *PD-L1* expression. In this study, *PD-L1* expression was higher in HCC cells than in normal liver cells. Overexpression of *PD-L1* reversed the effects on HCC cell proliferation, invasion, and colony formation caused by *HOXA-AS3* shRNA.

Nevertheless, this study had several limitations. Future studies should investigate whether *HOXA-AS3*/miR-455-5p alters the activity of CD8+ T cells via the PD-1/PD-L1 axis and whether targeting *HOXA-AS3* can increase the effectiveness of HCC immunotherapies based on PD-1/PD-L1 blockade.

## 5. Conclusions

Collectively, the results of this study revealed that *HOXA-AS3* expression was strikingly increased in HCC cells and that *HOXA-AS3* regulated *PD-L1* expression by sponging miR-455-5p. Additionally, overexpressed *HOXA-AS3* contributed to HCC cell invasion and proliferation by targeting the miR-455-5p/*PD-L1* axis. Therefore, *HOXA-AS3* may serve as a new target for HCC treatment and as a candidate biomarker for prognosis.

## Figures and Tables

**Table 1:** Sequences of primers for qRT-PCR.

Name		Sequence
HOXA-AS3	Forward	5′-AGGAAACATCAGGGCGTACA-3′
	Reverse	5′-ATCCTAAGTGCTTGCACCCT-3′

miR-455-5p	Forward	5′-ACACTCCAGCTGGGTATGTGCCTTTGGACT-3′
	Reverse	5′-CTCAACTGGTGTCGTGGAGTCGGCAATTCAGTTGAGCGATGTAG-3′

GAPDH	Forward	5′-AACGTGTCAGTGGTGGACCTG-3′
	Reverse	5′-AGTGGGTGTCGCTGTTGAAGT-3′

U6	Forward	5′-CTCGCTTCGGCAGCACA-3′
	Reverse	5′-AACGCTTCACGAATTTGCGT-3′

PD-L1	Forward	5′-TGGCATTTGCTGAACGCATTT-3′
	Reverse	5′-TGCAGCCAGGTCTAATTGTTTT-3′

## Data Availability

The data used to support the findings of this study are included within the article.

## Supplementary Materials

Supplementary Materials See Figure S1 in the Supplementary Material for comprehensive image analysis.
