# Supplementary figures and images for: HOXA-AS3 Promotes Proliferation and Migration of Hepatocellular Carcinoma Cells via the miR-455-5p/PD-L1 Axis
Source: J Immunol Res. 2021 Dec 27;2021:9289719. doi: 10.1155/2021/9289719 (PMC8723882; doi:10.1155/2021/9289719)

**A**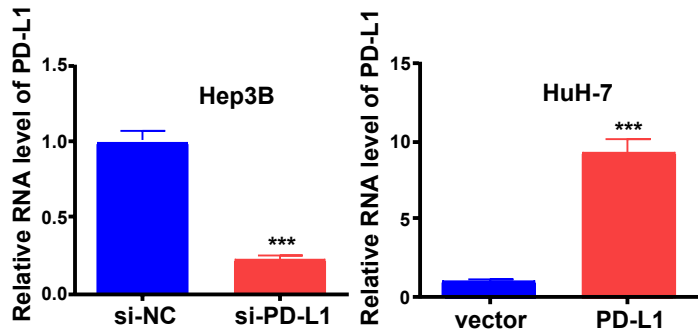**B**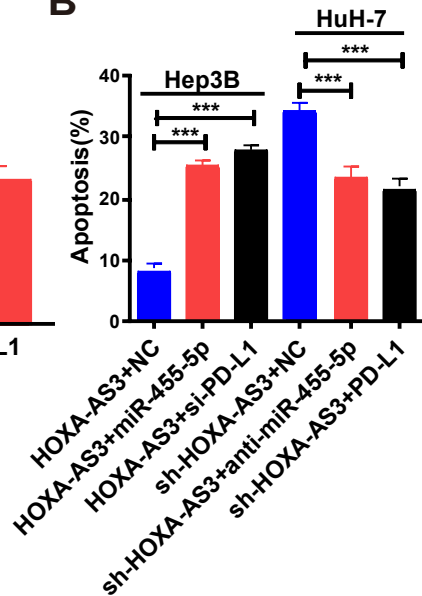**C**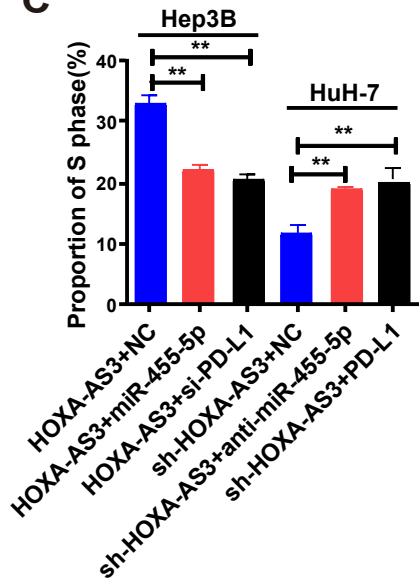

Supplement: Supplementary Materials — See Figure S1 in the Supplementary Material for comprehensive image analysis. [file 9289719.f1.pdf]
